# Pre‐referral general practitioner consultations and subsequent experience of cancer care: evidence from the English Cancer Patient Experience Survey

**DOI:** 10.1111/ecc.12353

**Published:** 2015-07-30

**Authors:** S.C. Mendonca, G.A. Abel, C.L. Saunders, J. Wardle, G. Lyratzopoulos

**Affiliations:** ^1^ Cambridge Centre for Health Services ResearchInstitute of Public HealthUniversity of CambridgeCambridgeUK; ^2^ RAND EuropeCambridgeUK; ^3^ Department of Epidemiology & Public HealthHealth Behaviour Research CentreUniversity College LondonLondonUK

**Keywords:** cancer, oncology, patient experience, referral, general practitioner, consultation

## Abstract

Prolonged diagnostic intervals may negatively affect the patient experience of subsequent cancer care, but evidence about this assertion is sparse. We analysed data from 73 462 respondents to two English Cancer Patient Experience Surveys to examine whether patients with three or more (3+) pre‐referral consultations were more likely to report negative experiences of subsequent care compared with patients with one or two consultations in respect of 12 *a priori* selected survey questions. For each of 12 experience items, logistic regression models were used, adjusting for prior consultation category, cancer site, socio‐demographic case‐mix and response tendency (to capture potential variation in critical response tendencies between individuals). There was strong evidence (*P* < 0.01 for all) that patients with 3+ pre‐referral consultations reported worse care experience for 10/12 questions, with adjusted odds ratios compared with patients with 1–2 consultations ranging from 1.10 (95% confidence intervals 1.03–1.17) to 1.68 (1.60–1.77), or between +1.8% and +10.6% greater percentage reporting a negative experience. Associations were stronger for processes involving primary as opposed to hospital care; and for evaluation than report items. Considering 1, 2, 3–4 and ‘5+’ pre‐referral consultations separately a ‘dose–response’ relationship was apparent. We conclude that there is a negative association between multiple pre‐diagnostic consultations with a general practitioner and the experience of subsequent cancer care.

## Background

Most cancer patients are diagnosed after the onset of symptoms caused by their cancer, typically after presenting to a general practitioner (GP; Elliss‐Brookes *et al*. 2012). Although most such patients are referred promptly for specialist assessment, some experience multiple consultations which lead to prolonged intervals to specialist referral (Lyratzopoulos *et al*. 2012, 2013). Policy initiatives in several countries aim to shorten intervals from presentation to diagnosis (Department of Health, 2001; Olesen *et al*. 2009; Prades *et al*. 2011). Several considerations motivate such policies, including improving clinical outcomes and minimising the frequency of medico‐legal complaints (Torring *et al*. 2012; Wallace *et al*. 2013). Furthermore, patients express a strong preference for prompt diagnostic assessment after presentation, and most would opt for investigation for possible cancer at risk levels as low as 1% (Pancreatic Cancer UK, 2011; Rarer Cancer Foundation, 2011; The Roy Castle Lung Cancer Foundation, 2011; Banks *et al*. 2014). It is therefore plausible that prolonged diagnostic intervals after presentation could be perceived by patients as indicative of sub‐optimal care early on in their journey, and negatively colour their experience of subsequent care. In the commercial sector, there is growing recognition of the enduring effects of the first encounter on subsequent service experience. Concordantly, surveys of cancer patients in the Netherlands and Denmark indicate that ‘rapid and adequate referral’ is one of the five most important aspects of care quality; and that delayed referral is associated with greater chance of decreased confidence in a patient's GP respectively (Larsen *et al*. 2011; Booij *et al*. 2013). Prior evidence also indicates that rapid diagnostic pathways may be associated with reduced patient anxiety (Brocken *et al*. 2014). Other evidence about the potential influence of diagnostic delays on the experience of subsequent cancer care is limited to case‐series with small sample sizes or is anecdotal (Risberg *et al*. 1996; Gallagher *et al*. 2010; Tomlinson *et al*. 2012). Indeed, a recent systematic review on the association between diagnostic timeliness and cancer outcomes lamented the lack of evidence of the impact of delays on patient‐reported outcomes and indicated ‘a dearth of studies reporting patient experience’ (Neal *et al*. 2015).

In recent years, large national surveys of cancer patients have been carried out in England (Cancer Patient Experience Survey, CPES). These include questions about the experience of several aspects of cancer care, including diagnostic testing, shared decision‐making, nurse communication, doctor communication, care coordination and overall satisfaction with cancer care. At the start of the questionnaire, respondents are also asked to indicate whether their diagnosis involved prior consultations with a GP, and if so, the number of such consultations. Against this background, we examined associations between the number of pre‐diagnostic GP consultations before referral for specialist assessment and the evaluation of subsequent cancer care.

## Methods

### Data

#### Source

We used anonymous data from respondents to the English CPESs 2011/2012 and 2012/2013 (hereafter referred to as ‘2012’ and 2013' surveys; Department of Health, 2012a, 2013). Both surveys were commissioned by the UK Department of Health and carried out by Quality Health (Chesterfield, UK), a specialist survey provider (Department of Health, 2012b; Quality Health, 2013). Items were cognitively tested in panels of volunteer patients, facilitated by a national cancer charity. The survey's sampling frame includes all patients treated in English National Health Service (NHS) hospitals for cancer during a 3‐month period (September to November 2011 and 2012, respectively for the 2012 and the 2013 surveys). After vital status checks, patients were mailed the survey questionnaire, with up to two reminders for non‐responders. Response rates were 68% and 64% for the 2012 and the 2013 surveys (Department of Health, 2012b; Quality Health, 2013). Anonymous data from the surveys are available for research purposes from the UK Data Archive ( http://www.data-archive.ac.uk/), as used in the present study (Department of Health, 2012a, 2013).

#### Sample derivation

For both surveys, information was available on patients' age, sex and International Classification of Diseases‐10 diagnosis code (based on hospital records); and self‐assigned ethnic group, using the Office of National Statistics 6‐category classification (based on responses to a survey item) (Saunders *et al*. 2013). We *a priori* restricted the analysis to patients who, in response to a survey item, had indicated that their cancer was diagnosed in the last year, to minimise potentially ‘double‐counting’ some respondents to the 2013 survey who might have also been sampled and responded to the 2012 survey. We also restricted the analysis to patients with any of 24 cancer diagnosis groups for which promptness of referral was previously described (Lyratzopoulos *et al*. 2012). We excluded from further analysis respondents with missing or non‐informative answers (‘don't know/can't say’) to questions 1 (on promptness of referral after presentation, the main exposure of prior interest, see below) and 70 (on overall care satisfaction with care, used in sensitivity analysis as explained below), and those with missing self‐assigned ethnicity, leaving 73,462 respondents for subsequent analyses (Appendix 1).

### Analysis

#### Main exposure variable

We used information from the survey question 1 ‘Before you were told you needed to go to hospital about cancer, how many times did you see your GP (family doctor) about the health problem caused by cancer?’, with possible informative answers ‘None – I did not see my GP before going to hospital’, ‘once’, ‘twice’, ‘three or four times’ and ‘five or more times’. For the main analysis, three categories of pre‐referral consultations with a GP before hospital referral were defined: 1 or 2 (‘1–2’); 3 or more (‘3+’); and no prior GP consultations.

#### Outcome variables

A group of 12 survey questions (items) was selected *a priori* to reflect different aspects of the cancer pathway across nine domains of care experience (Box 1). These included eight evaluative items (e.g. assessing the quality of inter‐personal care skills of nurses or doctors) and four items where patients reported on actual processes of care, such as whether they had access to a specialist nurse. These we termed report‐type items and, *a priori*, we did not expect associations with pre‐referral consultations. Of the 12 questions, three had binary response options and nine used a Likert response format. However, as public reporting conventions for the CPES use binary categories (positive/negative experience of care) for all questions, these binary forms were used in our analysis. Except for a single question (on length of waiting time to be seen as an outpatient) which was only included in one of the two surveys, all other 11 questions were (identically) included in both surveys – for ease of reference question numbers relate to the 2013 survey except if otherwise noted. The exact form of each question is provided in Box 1.

Box 1 **Exact wording of questions on aspects of care experience of cancer patients (question numbers correspond to the 2013 survey)**

**Table:** 

Questions (number, stem, questionnaire domain)
*Evaluation items*
12. *How do you feel about the way you were told you had cancer?* Within domain entitled ‘Finding out what was wrong with you’
20. *Were you involved as much as you wanted to be in decisions about your care and treatment?* Within domain ‘Deciding the best treatment for you’
38. *Did you have confidence and trust in the doctors treating you?* Within domain ‘Hospital Doctors’
42. *Did you have confidence and trust in the ward nurses treating you?* Within domain ‘Ward Nurses’
45. *While you were in hospital did you ever think that the doctors or nurses were deliberately not telling you certain things that you wanted to know?* Within domain ‘Hospital care and treatment’
64. *Do you think the GPs and nurses at your general practice did everything they could to support you while you were having cancer treatment?* Within domain ‘Care from your General Practice’
65. *Did the different people treating and caring for you (such as GP, hospital doctors, hospital nurses, specialist nurses, community nurses) work well together to give you the best possible care?* Within domain ‘Your overall NHS care’
70. *Overall, how would you rate your care?* Within domain ‘Your overall NHS care’
*Report items*
21. *Were you given the name of a Clinical Nurse Specialist who would be in charge of your care?* Within section entitled ‘Clinical Nurse Specialist’
53. *Were you given clear written information about what you should or should not do after leaving hospital?* Within domain ‘Hospital care and treatment’
61. (2012 survey). *The last time you had an outpatients appointment with a cancer doctor at one of the hospitals named in the covering letter, how long after the stated appointment time did the appointment start?* Within domain ‘Outpatient appointments with doctors’
63. *As far as you know, was your GP given enough information about your condition and the treatment you had at the hospital?* Within domain ‘Care from your General Practice’

#### Statistical analysis

For each of the 12 questions in turn, we used logistic regression models to examine associations between promptness of referral and subsequent care experience. After first describing crude proportions, we considered three separate models for each question, first estimating the crude (unadjusted) odds of negative experience; then the odds of negative experience adjusted for patient characteristics (age, sex and ethnicity) and cancer diagnosis; and lastly, the odds of negative experience adjusted for the overall response tendency of each individual patient, additionally to patient characteristics and cancer diagnosis. Response tendency is a construct often considered in patient‐reported outcome measures. It aims to capture potential variation in critical response tendencies between individuals. Adjusting for response tendency minimises the potential for apparent associations to be driven by common biases in the measurement of both the outcome (i.e. care experience) and exposure (i.e. number of consultations) variables by participants who provide answers that are systematically more or less critical than the average respondent. To create a measure of response tendency for each patient, we adjusted their responses to each individual question for their answers to up to nine other questions as detailed in Appendix 2. Essentially, this approach adjusts the reported experience for clustering of more or less critical responses among individual respondents.

#### Supplementary analysis

In supplementary analysis, we examined the presence of a ‘dose–response’ relationship (i.e. whether greater number of consultations was associated with less positive experience). We did this by considering all four ordinal categories of pre‐referral consultations included as possible responses in the relevant survey item separately (i.e. ‘once’, ‘twice’, ‘three or four times’, and ‘five or more times’).

#### Sensitivity analysis

We repeated the main analysis model additionally adjusting for patient socioeconomic status, based on the Index of Multiple Deprivation 2007 scores of the lower super output area of patients' residence (only available for 2013 survey respondents) (Indices of Deprivation, 2007). In addition, for each of the 11 questions other than overall satisfaction (question 70), we repeated the logistic regression model by substituting the measure of response tendency described above with the patient's overall satisfaction with their care.

## Results

### Sample description

Among 73 462 patients included in the initial analysis sample, 44 827 (61.0%) had seen their GP once or twice before referral and 13 280 (18.1%) had seen the GP three or more times, while in 15 355 (20.9%) patients, the diagnostic process did not involve prior consultation with a GP. Among patients whose diagnosis involved at least one primary care consultation, 77.1% had seen their GP once or twice, and 22.9% three or more times. The number of patients with valid responses to each of the 12 outcome questions ranged from 32 999 (for question 61, regarding length of waiting time in the outpatient department; a question only included in the 2013 survey) to 73 452 (for question 70, overall satisfaction with cancer care). The variability in the number of respondents by question chiefly reflects the fact that some questions do not apply to all patients (e.g. the question on confidence and trust towards ward nurses would only apply to patients who had an inpatient stay during the sampling period). Crude proportions of patients reporting a negative experience varied substantially between questions, from 5.3% of patients reporting that their GP was not given enough information about their treatment plan (question 63) to 34.5% of patients indicating sub‐optimal coordination of their care (question 65). The analysis sample comprised patients with 24 different diagnosis of cancer, the three most common cancers being breast (18 787, 26%), colon (7357, 10%) and prostate (6180, 8%) whilst the three most rarer were laryngeal (521, 0.7%), testicular (441, 0.6%) and vulval (248, 0.3%), Appendix 3.

### Consultations and subsequent care experience

Patients with three or more consultations were more likely (*P* < 0.001) to report a negative experience than patients with only one or two consultations for all 12 questions, with odds ratios ranging from 1.17 to 1.91 (Tables 1 and 2).

**Table 1:** Sample size, observed (crude) proportion of patients reporting a negative experience, and crude and adjusted percentage of negative experience, by question

Question (number and synoptic form)		Experience domain	N	Overall % negative experience	Crude % negative experience by number of pre‐referral consultations		% crude difference in negative experience (‘3+’ –‘1–2’)	Adjusted % negative experience by number of pre‐referral consultations^a^		% adjusted difference in negative experience (‘3+’ – ‘1–2’)
					‘1–2’	‘3+’		‘1–2’	‘3+’	
*Evaluation items*										
12	Told diagnosis sensitively	Told had cancer	72 621	14.9	13.5	21.5	7.9	14.0	18.0	4.0
20	Shared decision‐making	Decision‐making	70 269	25.8	24.8	31.5	6.8	25.5	27.6	2.1
38	Confidence and trust in hospital doctors	Hospital doctors	55 494	13.2	12.1	18.9	6.7	12.9	14.7	1.8
42	Confidence and trust in ward nurses	Ward nursing	55 229	28.8	27.2	36.0	8.9	28.1	31.7	3.5
45	Thought information withheld from them	Hospital care	55 294	10.7	9.7	16.3	6.6	10.2	12.3	2.0
70	Overall care satisfaction	Overall care	73 452	10.8	9.3	16.4	7.1	9.9	12.7	2.8
64	General practice staff support	Primary care	49 158	31.0	27.2	41.0	13.8	27.9	38.5	10.6
65	Cancer care integration	Overall care	70 003	34.5	31.8	44.7	12.9	32.9	40.0	7.1
*Report items*										
21	Given name of clinical nurse specialist (CNS)	Specialist nursing	69 793	9.2	9.1	10.5	1.4	9.0	9.0	0.0
53	Written information at discharge	Hospital care	52 355	14.4	13.5	19.5	6.0	14.2	14.8	0.6
61	Outpatient appointment waiting time	Outpatient care	32 999	28.6	27.8	31.1	3.3	28.3	30.1	1.8
63	Information given to GP (by hospital)	Primary care	59 073	5.3	4.5	8.0	3.5	4.7	6.2	1.5

a Predicted probabilities derived from logistic regression models adjusted for response tendency using a response tendency measure (see Methods, statistical analysis).

**Table 2:** Odds ratios (and related 95% confidence intervals and *P*‐values) for negative experience between patients with ‘three or more consultations’ with a general practitioner compared with ‘one or two consultations’ (used as the reference category)

Question (number and synoptic form)	N	Unadjusted (crude) odds ratios			Odds ratios adjusted for patient case‐mix (i.e. age, sex, ethnicity and cancer diagnosis)			Odds ratios adjusted for patient case‐mix and measure of response tendency		
		Odds ratio	95% confidence interval	P	Odds ratio	95% confidence interval	P	Odds ratio	95% confidence interval	P
*Evaluation items*										
Q64 Practice staff support	49 158	1.86	1.77–1.95	<0.001	1.84	1.75–1.93	<0.001	1.68	1.60–1.77	<0.001
Q65 Cancer care integration	70 003	1.74	1.67–1.81	<0.001	1.67	1.60–1.74	<0.001	1.48	1.41–1.55	<0.001
Q70 Overall care satisfaction	73 452	1.91	1.81–2.02	<0.001	1.77	1.67–1.88	<0.001	1.44	1.35–1.54	<0.001
Q12 Told diagnosis sensitively	72 621	1.75	1.66–1.84	<0.001	1.53	1.46–1.62	<0.001	1.38	1.31–1.46	<0.001
Q45 Thought information withheld	55 294	1.82	1.71–1.95	<0.001	1.53	1.42–1.63	<0.001	1.27	1.18–1.37	<0.001
Q42 Confidence and trust – ward nurse	55 229	1.51	1.44–1.59	<0.001	1.40	1.33–1.47	<0.001	1.22	1.15–1.29	<0.001
Q38 Confidence and trust – hospital doctor	55 494	1.69	1.59–1.79	<0.001	1.52	1.42–1.62	<0.001	1.22	1.13–1.31	<0.001
Q20 Shared decision‐making	70 269	1.40	1.34–1.46	<0.001	1.32	1.26–1.38	<0.001	1.13	1.08–1.19	<0.001
*Report items*										
Q63 Information given to GP	59 073	1.85	1.70–2.02	<0.001	1.64	1.50–1.79	<0.001	1.36	1.24–1.50	<0.001
Q61 Outpatient waiting time	32 999	1.17	1.10–1.25	<0.001	1.17	1.10–1.25	<0.001	1.10	1.03–1.17	0.007
Q53 Written info post‐discharge	52 355	1.55	1.46–1.65	<0.001	1.27	1.20–1.36	<0.001	1.06	0.99–1.13	0.100
Q21 Given name of Clinical Nurse Specialist (CNS)	69 793	1.18	1.10–1.26	<0.001	1.10	1.03–1.18	0.006	1.00	0.93–1.07	0.894

Questions are ordered by effect size for response tendency model within evaluation and report categories.

After adjusting for age, sex, ethnicity and cancer diagnosis, there was strong evidence (*P* < 0.01 for all) that reported negative experience was more common among patients with three or more consultations compared with those who had just one or two consultations across all 12 questions (Table 2). There was a degree of attenuation of effect sizes (i.e. adjusted odds ratio values being lower compared with unadjusted odds ratio ones), indicating that crude associations were partially confounded by patient characteristics and cancer diagnosis.

**Figure 1:**
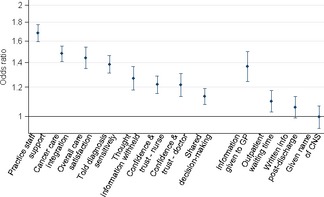
Odds ratios (and 95% CIs) for negative experience for patients with ‘three or more’ pre‐referral consultations with a general practitioner, compared with patients with 1–2 consultations (reference). Questions ordered by effect size with evaluative questions on the left and report questions on the right. CI, confidence interval.

After additionally adjusting for response tendency, there was still evidence that patients with three or more pre‐referral consultations were more likely to report negative experience for 10 of the 12 questions (*P* ≤ 0.007), with a degree of further attenuation of effect sizes (Table 2; Fig. 1).

Generally, observed associations tended to be stronger for the evaluative‐type questions which reflected aspects of management that at least partially involve primary care, e.g. the degree of support provided to cancer patients by staff in their general practice (question 64), and the experience of integration between hospital and primary care (question 65). In contrast, for report‐type questions solely relating to within‐hospital care processes (e.g. outpatient's waiting time, or access to specialist nursing, questions 61 and 21 respectively) associations were weak (Tables 1 and 2; Fig. 1).

To further illustrate the findings, we used the outputs of the fully adjusted (i.e. for patient case‐mix and response tendency) regression models used in the main analyses to calculate the predicted percentage of patients reporting negative experiences should all patients had been in each of the different categories of number of consultations. Compared with patients with 1–2 consultations, those with 3+ consultations had between +1.8% and +10.6% greater absolute proportions of negative experience, for the 10 questions with a significant association (Table 1).

### Supplementary and sensitivity analyses

**Figure 2:**
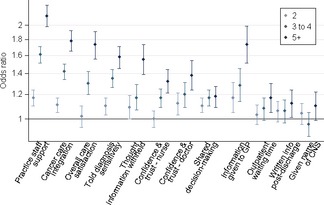
Supplementary analysis considering the odds of negative experience, by number of pre‐referral consultations (‘one’ pre‐referral consultation used as the reference category). Note overall clear ‘dose–response’ relationship for questions where evidence of association is present (see Table 2 and main text). Questions ordered as in Figure 1.

Considering each ordinal category of the number of pre‐referral consultations separately, a strong ‘dose–response’ monotonic pattern was apparent, with greater number of consultations consistently associated with greater chance of reported negative evaluation of experience (Fig. 2, Appendix 4). Adjustment for socioeconomic status (2013 survey sample only) produced findings that were concordant with those observed in the main analysis. Adjusting for overall satisfaction with care (question 70) as an alternative measure of response tendency produced similar findings with those observed in the main analysis, uniformly for the 11 questions where this analysis was applicable, with odds ratio values between those obtained by the main analysis (adjusted for both patient case‐mix and a measure of response tendency) and those adjusted for patient case‐mix alone (Appendix 5).

## Discussion

The findings indicate that patients with cancer are more likely to report worse care experience if they had a greater number of pre‐diagnosis consultations with a GP before they were referred for specialist assessment. These associations are particularly apparent for evaluation (as opposed to report) items that at least partially reflect aspects of post‐diagnosis management that involve primary care staff. The findings were robust to sensitivity analyses adjusting for patient deprivation status and different approaches to measuring response tendency, and showed a clear ‘dose–response’ pattern.

### Findings in relation to other evidence

Prior evidence about the impact of promptness of investigation after symptomatic presentation on care experience comes from smaller surveys restricted to a much narrower spectrum of aspects of care experience (Risberg *et al*. 1996; Larsen *et al*. 2011; Tomlinson *et al*. 2012; Booij *et al*. 2013). A number of cancer charities have in recent years advocated that delays in suspecting cancer in primary care may have a negative impact on care experience for patients and their loved ones (Pancreatic Cancer UK, 2011; Rarer Cancer Foundation, 2011; The Roy Castle Lung Cancer Foundation, 2011). In addition, patients express strong preferences for timely investigation for suspected cancer (Banks *et al*. 2014). The findings therefore substantially augment the present state of evidence, and at least partially address a recent call by authors of a systematic review for more evidence on the association between timeliness of diagnosis and patient‐reported outcome measures (Neal *et al*. 2015).

To further contextualise the findings, it is useful to indirectly compare the size of observed differences to that of other, previously, described variations in the experience of cancer care, e.g. variations by age group or cancer site. In that respect, the odds of negative experience for patients with 3+ consultations for question 64 (on practice staff support, i.e. the item with the largest noted difference and an adjusted odds ratio value of 1.68) is of similar magnitude to differences in patient experience for the same item between 25–34 and 65–74 year olds, or between patients with renal and rectal cancer (Saunders *et al*. 2015). These comparisons indicate that overall the differences cannot be dismissed as ignorable, especially if we consider that this binary categorisation (three or more vs. one or two consultations) hide larger differences between extreme categories (i.e. 5+ consultations vs. 1 consultation – see Appendix 4).

### Strengths and limitations

The study strengths include its large nationwide sample, and the inclusion of patients diagnosed in a recent period. Furthermore, we were able to adjust the analyses for socio‐demographic characteristics and cancer diagnosis, variables known to be associated with both promptness of specialist referral and the evaluation of care experience (Lyratzopoulos *et al*. 2012, 2013; Saunders *et al*. 2015). In addition, we have been able to adjust the findings for potential bias from differential response tendency. The survey also had a relatively high (for a postal questionnaire survey) response rate, with about two‐thirds of eligible patients responding. For comparisons, the large Hospital Consumer Assessment and Healthcare Providers and Systems survey of US patients has a response rate of <40%, as does the English General Practice Patient Survey (Jha *et al*. 2008; Roland *et al*. 2009). Furthermore, the fact that estimates of associations were case‐mix adjusted minimises concerns about potential bias measurement of these associations (Groves & Peytcheva 2008).

Another limitation is that by the nature of the study we were not able to examine the potential influence of a range of variables, which may confound or/and mediate the observed association. For example, considering potential confounding, cancer patients with a higher level of co‐morbidity may both report more critical experiences (because of greater care needs) and be at higher risk of multiple pre‐referral consultations (if symptoms caused by their cancer are wrongly attributed to their pre‐existing conditions). In addition, some patients may have personality traits which may impede the effectiveness of communication with a doctor during a consultation and at the same time be associated with a tendency to respond to experience questions more critically. Furthermore, considering potential mediators, for patients who present with organ‐confined tumours, untimely referral may increase the risk progression to a more advanced stage, which is in turn associated with worse experience of care (Ayanian *et al*. 2010). We do not, however, believe this is likely to be a mechanism affecting more than very few patients in our sample, given the fact that, on average, delays associated with greater number of consultations are relatively pretty short on average (i.e. an approximate median of 1 and 1.5 months for patients who experience three or four pre‐referral consultations) (Lyratzopoulos *et al*. 2013). Critically, the above limitations need to be interpreted in the light of the fact that observed associations are more pronounced for aspects of management that involve primary care, and tend to be concentrated on evaluative as opposed to report items. These observations indicate that where present, associations between promptness of referral and subsequent care experience cannot be fully explained by potential either residual confounding or the mediating effects of disease progression.

We were not able to directly measure the impact of additional number of pre‐referral consultations on the overall length of the primary care interval (i.e. the number of days from presentation to referral) (Weller *et al*. 2012). However, national audit evidence indicates that the number of pre‐referral consultations is strongly associated with the length of the primary care interval (Spearman's rank correlation coefficient *r* = 0.70) (Lyratzopoulos *et al*. 2013). Specifically, while the median primary care interval for patients with a single consultation is 0 days, it is 34, 47 and 96 days for patients with three, four and five or more consultations respectively (Lyratzopoulos *et al*. 2013). Therefore, the measure used in this study (number of consultations) has construct validity as a marker of the length of the primary care interval. We had no information on symptoms at presentation, and we were therefore unable to examine potential variation in the observed associations by symptom type and/or adjust for symptom status. Further, associations between untimely referral and subsequent experience may differ between patients with different presenting symptoms, a question for future research.

### Interpretation and implications

The findings indicate that multiple pre‐referral consultations with a GP seem to ‘prime’ patients for a less positive evaluation of the experience of subsequent care. Therefore, they provide an additional supportive argument for policy initiatives and ongoing research aimed at reducing diagnostic delays after symptomatic presentations in primary care. These initiatives may include diagnostic test development and greater use of existing tests; decision‐making support during the primary care consultation; and system‐wide engineering approaches (such as enabling greater access to specialist advice and investigations) (Lyratzopoulos *et al*. 2014).

Furthermore, the fact that untimely diagnosis may affect the experience of post‐diagnosis cancer management in primary care has implications for survivorship care, given the increasing development of care models embedded in general practice (Emery 2014).

Future research should aim to examine whether co‐morbidity and stage at diagnosis confound or mediate the observed association between promptness of referral and subsequent experience. Such analysis can be ideally supported by examining patient survey and clinical outcomes considered together. Future studies should also assess the potential impact of less prompt referral on the quality of life of cancer patients and the psychological mechanisms by which it affects subsequent experience (Robinson *et al*. 2012). Qualitative studies of cancer patients with prompt and untimely referral history would be highly valuable.

We should lastly state that, in itself, the number of pre‐referral consultations is a measure of experience. Therefore, it cannot be argued that number of consultations would have not mattered for patient experience had null associations between number of pre‐referral consultations and *subsequent* aspects of experience been observed.

In conclusion, we have provided large scale evidence from a real‐world setting suggesting that less prompt referral for specialist assessment after symptomatic presentation negatively affect the experience of subsequent cancer care. These realisations support efforts to increase the proportion of cancer patients who experience a prompt referral.

## Conflicts of interest

The authors have no conflicts of interest to declare.

## Appendix 2 Calculation of response tendency measure

For this analysis, the data were set in a ‘long’ format. In such a dataset, each patient has a separate record for the response to each question (i.e. multiple records per patient). We estimated response tendency by running mixed effect models predicting the odds of negative experience across all questions, adjusting for question number with a fixed effect, and with a random intercept for patient. The fixed effect for question number accounts for the fact that some questions are more likely than others to be answered positively by all patients and the random effect for patient accounts for the fact that responses to multiple questions are clustered within patients and that some patients are more likely to give negative responses (regardless of question number) than others. This random effect can be considered to capture a latent variable which is the patients underlying response tendency. To obtain an estimate of this latent variable for each patient we calculate the best linear unbiased predictor for the random effect, and used this as our response tendency variable in the main analysis.

We obtained nine different versions of this latent variable, depending on the outcome question of the multinomial model. In all models, we excluded from adjustment answers to the three questions that related to processes of care which may have involved primary care staff (because of potential for an intrinsic association between primary care experience and primary care diagnosis); and the question about overall satisfaction with cancer care (both because satisfaction may be influenced by primary care processes, and because it is a possible marker of response tendency in itself). For these four questions we used a common latent variable. For the remaining eight outcome questions we excluded adjusting for that question from the random effect model. The following table indicates the adjustments made for each outcome question.

**Table:**

	Outcome in main analysis model	Questions excluded from random effect model
12	Told diagnosis sensitively	12 and 63, 64, 65, 70
20	Shared decision‐making	20 and 63, 64, 65, 70
21	Given name of clinical nurse specialist (CNS)	21 and 63, 64, 65, 70
38	Confidence and trust in doctors	38 and 63, 64, 65, 70
42	Confidence and trust in ward nurses	42 and 63, 64, 65, 70
45	Thought information withheld from them	45 and 63, 64, 65, 70
53	Written information at discharge	53 and 63, 64, 65, 70
61	Outpatient appointment waiting time	61 and 63, 64, 65, 70
63	Information given to GP (by hospital)	63, 64, 65, 70
64	General practice staff support	63, 64, 65, 70
65	Cancer care integration	63, 64, 65, 70
70	Overall care satisfaction	63, 64, 65, 70

## Appendix 3 Sample composition in terms of cancer diagnosis

**Table:**

	*N*	%
Breast	18 787	25.6
Colon	7357	10
Prostate	6180	8.4
Lung	5589	7.6
Bladder	5532	7.5
Rectal	4801	6.5
Non‐Hodgkin lymphoma	4445	6.1
Melanoma	2558	3.5
Endometrial	2402	3.3
Oesophageal	2173	3
Ovarian	2077	2.8
Multiple myeloma	1812	2.5
Leukaemia	1743	2.4
Stomach	1460	2
Renal	1276	1.7
Pancreatic	906	1.2
Hodgkin's lymphoma	689	0.9
Thyroid	686	0.9
Cervical	664	0.9
Brain	569	0.8
Mesothelioma	546	0.7
Laryngeal	521	0.7
Testicular	441	0.6
Vulval	248	0.3

## Appendix 4 Supplementary analysis considering all five categories of number of pre‐referral consultations available

**Table:**

	Question	Number of pre‐referral visits	Odds ratio^b^	95% confidence interval	*P* a
Evaluation questions	Practice staff support	1	1.00	–	<0.001
		2	1.17	1.10–1.24	
		3–4	1.61	1.51–1.71	
		5+	2.14	1.98–2.32	
	Cancer care integration	1	1.00	–	<0.001
		2	1.11	1.05–1.17	
		3–4	1.42	1.34–1.50	
		5+	1.78	1.65–1.92	
	Overall care satisfaction	1	1.00	–	<0.001
		2	1.02	0.94–1.10	
		3–4	1.30	1.20–1.42	
		5+	1.73	1.56–1.91	
	Told diagnosis sensitively	1	1.00	–	<0.001
		2	1.10	1.03–1.17	
		3–4	1.35	1.26–1.44	
		5+	1.58	1.45–1.71	
	Thought information withheld	1	1.00	–	<0.001
		2	1.09	1.00–1.19	
		3–4	1.17	1.07–1.29	
		5+	1.55	1.39–1.73	
	Confidence and trust – nurse	1	1.00	–	<0.001
		2	1.00	0.94–1.06	
		3–4	1.17	1.09–1.25	
		5+	1.32	1.21–1.44	
	Confidence and trust – doctor	1	1.00	–	<0.001
		2	1.12	1.03–1.21	
		3–4	1.20	1.09–1.31	
		5+	1.38	1.24–1.54	
	Shared decision‐making	1	1.00	–	<0.001
		2	1.10	1.05–1.17	
		3–4	1.17	1.10–1.24	
		5+	1.18	1.09–1.27	
Report questions	Information given to GP	1	1.00	–	<0.001
		2	1.17	1.05–1.31	
		3–4	1.28	1.14–1.45	
		5+	1.73	1.51–1.99	
	Outpatient waiting time	1	1.00		0.020
		2	1.03	0.96–1.11	
		3–4	1.08	0.99–1.17	
		5+	1.17	1.05–1.30	
	Written info post‐discharge	1	1.00	–	0.123
		2	1.06	0.98–1.14	
		3–4	1.06	0.97–1.15	
		5+	1.12	1.01–1.24	
	Given name of CNS	1	1.00	–	0.133
		2	1.04	0.96–1.12	
		3–4	0.96	0.88–1.05	
		5+	1.10	0.99–1.22	

Odds ratio values relate to negative experience outcomes. Questions are ordered as in Table 2 (main text).

a From joint tests for the categories ‘twice’, ‘three or four times’ and ‘five or more times’.

b Adjusted for age group, sex, ethnicity, cancer diagnosis and measure of response tendency.

## Appendix 5 Sensitivity analysis using an alternative approach to measuring response tendency (using response to question 70 – overall satisfaction with cancer care)

**Table:**

Question (number and synoptic form)	*N*	Odds ratios adjusted for patient case‐mix (age, sex, ethnicity and cancer diagnosis) ratios^a^			Odds ratios adjusted for patient case‐mix and response to Question 70 (as an alternative measure of response tendency)			Odds ratios adjusted for patient case‐mix and measure of response tendency^a^		
		Odds ratio	95% CI	*P*	Odds ratio	95% CI	*P*	Odds ratio	95% CI	*P*
*Evaluation items*										
Q64 Practice staff support	49 158	1.84	1.75–1.93	<0.001	1.72	1.64–1.81	<0.001	1.68	1.60–1.77	<0.001
Q65 Cancer care integration	70 003	1.67	1.60–1.74	<0.001	1.53	1.46–1.61	<0.001	1.48	1.41–1.55	<0.001
Q70 Overall care satisfaction	73 452	1.77	1.67–1.88	<0.001	n/a			1.44	1.35–1.54	<0.001
Q12 Told diagnosis sensitively	72 621	1.53	1.46–1.62	<0.001	1.40	1.33–1.48	<0.001	1.38	1.31–1.46	<0.001
Q45 Thought information withheld	55 294	1.53	1.42–1.63	<0.001	1.33	1.23–1.43	<0.001	1.27	1.18–1.37	<0.001
Q42 Confidence and trust – ward nurse	55 229	1.40	1.33–1.47	<0.001	1.26	1.20–1.33	<0.001	1.22	1.15–1.29	<0.001
Q38 Confidence and trust – hospital doctor	55 494	1.52	1.42–1.62	<0.001	1.29	1.20–1.38	<0.001	1.22	1.13–1.31	<0.001
Q20 Shared decision‐making	70 269	1.32	1.26–1.38	<0.001	1.17	1.12–1.23	<0.001	1.13	1.08–1.19	<0.001
*Report items*										
Q63 Information given to GP	59 073	1.64	1.50–1.79	<0.001	1.41	1.28–1.55	<0.001	1.36	1.24–1.50	<0.001
Q61 Outpatient waiting time	32 999	1.17	1.10–1.25	<0.001	1.11	1.04–1.19	0.002	1.10	1.03–1.17	0.007
Q53 Written info post‐discharge	52 355	1.27	1.20–1.36	<0.001	1.13	1.06–1.21	<0.001	1.06	0.99–1.13	0.100
Q21 Given name of Clinical Nurse Specialist (CNS)	69 793	1.10	1.03–1.18	0.006	1.02	0.95–1.10	0.556	1.00	0.93–1.07	0.894

Odds ratios (and related 95% confidence intervals and *p*‐values) for negative experience between patients with ‘three or more consultations’ with a general practitioner compared with ‘one or two consultations’ (used as the reference category). Questions are ordered by effect size for response tendency model within evaluation and report categories.

a Reproduced from Table 2 – presented here for ease of comparisons.
